# Significance of a histone-like protein with its native structure for the diagnosis of asymptomatic tuberculosis

**DOI:** 10.1371/journal.pone.0204160

**Published:** 2018-10-25

**Authors:** Yukiko Ohara, Yuriko Ozeki, Yoshitaka Tateishi, Tsukasa Mashima, Fumio Arisaka, Yasuo Tsunaka, Yoshie Fujiwara, Akihito Nishiyama, Yutaka Yoshida, Kengo Kitadokoro, Haruka Kobayashi, Yukihiro Kaneko, Ichiro Nakagawa, Ryoji Maekura, Saburo Yamamoto, Masato Katahira, Sohkichi Matsumoto

**Affiliations:** 1 Department of Bacteriology, Niigata University School of Medicine, Niigata, Japan; 2 Department of Microbiology, Kyoto University Graduate School of Medicine, Kyoto, Kyoto, Japan; 3 Graduate School of Energy Science, Kyoto University, Gokasho, Uji, Kyoto, Japan; 4 College of Bioresource Sciences, Nihon University, Fujisawa, Kanagawa, Japan; 5 Graduate School of Medical Life Science, Yokohama City University, 1-7-29 Suehiro-cho, Tsurumi-ku, Yokohama, Japan; 6 Institute for Integrated Cell-Material Sciences (WPI-iCeMS), Kyoto University, Yoshida-Honmachi, Sakyo-ku, Kyoto, Japan; 7 Department of Structural Pathology, Institute of Nephrology, Graduate School of Medicine, Niigata University, Niigata, Japan; 8 Graduate School of Science and Technology, Department of Biomolecular Engineering, Kyoto Institute of Technology, Matsugasakigosyokaido-cho, Sakyo-ku, Kyoto, Japan; 9 Department of Bacteriology and Virology, Osaka-City University Graduate School of Medicine, Osaka, Japan; 10 Department of Respiratory Medicine, National Hospital Organization Toneyama National Hospital, 5-1-1 Toneyama, Toyonaka, Osaka, Japan; 11 Graduate School of Health Care Sciences, Jikei Institute, Osaka, Japan; 12 Central Laboratory, Japan BCG Laboratory, Kiyose-shi, Tokyo, Japan; University of Padova, Medical School, ITALY

## Abstract

Tuberculosis causes the highest mortality among all single infections. Asymptomatic tuberculosis, afflicting one third of the global human population, is the major source as 5–10% of asymptomatic cases develop active tuberculosis during their lifetime. Thus it is one of important issues to develop diagnostic tools for accurately detecting asymptomatic infection. Mycobacterial DNA-binding protein 1 (MDP1) is a major protein in persistent *Mycobacterium tuberculosis* and has potential for diagnostic use in detecting asymptomatic infection. However, a previous ELISA-based study revealed a specificity problem; IgGs against MDP1 were detected in both *M*. *tuberculosis*-infected and uninfected individuals. Although the tertiary structures of an antigen are known to influence antibody recognition, the MDP1 structural details have not yet been investigated. The N-terminal half of MDP1, homologous to bacterial histone-like protein HU, is predicted to be responsible for DNA-binding, while the C-terminal half is assumed as totally intrinsically disordered regions. To clarify the relationship between the MDP1 tertiary structure and IgG recognition, we refined the purification method, which allow us to obtain a recombinant protein with the predicted structure. Furthermore, we showed that an IgG-ELISA using MDP1 purified by our refined method is indeed useful in the detection of asymptomatic tuberculosis.

## Introduction

Tuberculosis remains a serious threat to human health. The most recent report by the World Health Organization announced that, in 2016, 10.4 million people newly developed tuberculosis and 1.7 million people died from this disease[1]. *Mycobacterium tuberculosis*, an acid-fast Gram-positive bacillus, is an etiologic agent of tuberculosis. This intracellular pathogen can escape from the bactericidal mechanisms of its hosts. It is estimated that more than 90% of *M*. *tuberculosis*-infected individuals do not initially develop the disease but instead remain in an asymptomatic state without eradicating the pathogen[2, 3].

Notably, reactivation of the disease occurs in 5–10% of these asymptomatic cases during their lifetimes. Thus, asymptomatic infections are the major silent source of tuberculosis. The establishment of a precise diagnosis of asymptomatic tuberculosis is thus crucial for disease control[3, 4]. However, the sensitivity of currently available tools, such as the interferon-gamma release assay (IGRA), is limited, probably because they detect immune responses to proteins produced from growing rather than from persistent *M*. *tuberculosis*[5, 6].

The majority of persistent *M*. *tuberculosis* is thought to exist in the stationary or dormant phase. Utilization of the antigens produced by persistent *M*. *tuberculosis* is a rational approach to the development of a diagnosis method for asymptomatic tuberculosis. Mycobacterial DNA-binding protein 1 (MDP1) is a major cellular protein of *M*. *tuberculosis*, and its expression is enhanced during both the stationary and dormant phases, in turn causing growth suppression [7–10]. MDP1 also induces the tolerance to isoniazid, a front line anti-tuberculosis drug [11], which is problemic characteristics of dormant *M*. *tuberculosis* [2, 12]. The expression of MDP1 can be triggered by an iron deficiency[13, 14], which mimics intracellular environments. These reports suggest that individuals with asymptomatic tuberculosis have substantial levels of MDP1 expression. In fact, anti-MDP1 antibodies stained a lung biopsy sample derived from a person who had not developed tuberculosis[15].

Both the IgG and T-cell responses to MDP1 are elevated in patients with asymptomatic tuberculosis, such as latent tuberculosis infection (LTBI) and past tuberculosis compared with that in patients with active tuberculosis [15, 16]. In contrast, both B- and T-cell immune responses to other tested antigens, such as early secretary antigen target with 6 kDa (ESAT6), culture filtrate protein 10 kDa (CFP10) [17], and alpha-crystalline-like protein (Acr or HspX)[18] are higher in active tuberculosis patients than in patients with LTBI or past tuberculosis[15, 16]. Taken together, these data suggest that MDP1 is an antigenic marker for asymptomatic infection.

Antibodies can recognize both the primary and tertiary structures of proteins. The N-terminal half of MDP1 has homology with the bacterial histone-like protein HU, while the C-terminal half is a eukaryotic histone-like region containing repetitive sequences rich in lysine, alanine, and proline. The crystal structure of the N-terminal half of MDP1 was shown to form a HU-like dimer with long symmetric arms that is presumably responsible for DNA-binding[19]. Our present sequence analyses suggest that the C-terminal half should be classified as intrinsically disordered regions (IDRs). Thus, MDP1 is an intrinsically disordered protein (IDP) like eukaryotic histones, which are rare in bacteria[20].

It is crucial to develop a method that allows us to obtain MDP1 with its native structure that is recognized by the antibodies produced during natural *M*. *tuberculosis* infection. In previous studies, we purified MDP1 by using acid extraction (AC-rfull-MDP1), based on a method commonly used for the purification of eukaryotic histones[21, 22], and applied it to an IgG enzyme-linked immunosorbent assay (ELISA)[10, 15, 16]. However, acid extraction is likely to have caused MDP1 denaturation. In this study, we refined the purification method to work without acid extraction and examined the antigenicity of MDP1 to IgG produced in *M*. *tuberculosis*-infected individuals.

## Materials and methods

### Structure prediction of MDP1

The secondary structure of MDP1 was predicted by the GTOP program of the National Institute of Genetics, Japan.

### Recombinant protein preparation

*E*. *coli* BL21 (DE3) pLysS (L1191, Promega, Madison, WI, USA) cells were transformed with the previously constructed plasmid that expresses rFull-MDP1[23]. The next day, a single colony was inoculated in 40 mL of LB media and cultured at 37 °C with shaking. This initial small culture was then transferred into 4 L of fresh LB media and further cultured at 37 °C with shaking at 100 rpm/min for approximately 2 h until reaching an optical density at 600 nm of 0.6. Isopropylβ-D-1-thiogalactopyranoside (IPTG) was then added to a final concentration of 0.5 mM, and the bacteria were further cultured for 3 h at 37 °C with shaking at the same speed. At the end of the incubation, the culture was immediately cooled on ice and then centrifuged at 7,000 rpm for 10 min at 4 °C using a RA-8R rotor (KUBOTA 7800). After removing the supernatant, the pellet was washed with ice-cold phosphate-buffered saline (PBS), and the bacteria were subsequently collected by centrifugation. The cells were re-suspended in 45 mL of buffer A (50 mM sodium phosphate [pH 7.4], 100 mM sodium chloride, 0.2 mM EDTA, and 0.1 mM phenylmethanesulfonyl fluoride [PMSF]) per 1 L of culture and then disrupted by using an ultrasonic generator with cooling on ice. After removing clumps of disrupted lysate with a combination of centrifugation and filtration using a membrane filter with a pore size of 0.22 μm, the bacterial lysate was loaded onto a His Trap column (bed volume, 5 mL; 17524802 GE Healthcare) and eluted with a linear gradient of 10–300 mM imidazole in buffer A. The proteins in the eluted fractions were analysed by SDS-PAGE using a 15% polyacrylamide gel.

The protein fractions containing rFull-MDP1 were then precipitated with ammonium sulfate with stirring on ice for 3 h to produce a saturation degree of 80%. The sample was subsequently dialyzed at 4 °C overnight in a buffer containing 50 mM sodium phosphate, 300 mM sodium chloride, and 10 mM imidazole (pH 6.8) and applied to a heparin column (HiTrap Heparin HP 17040701, GE Healthcare). The proteins were eluted with a linear gradient of 300–1,500 mM NaCl. After checking the fractions that contained the eluted rN-MDP1 by SDS-PAGE, the fractions were dialyzed overnight at 4 °C in buffer containing 50 mM sodium phosphate, 300 mM sodium chloride, and 10 mM imidazole (pH 6.8). The proteins were then loaded onto a CM Sepharose column (HiTrap CM FF 17515501, GE Healthcare) and eluted with a linear gradient of 300–1,500 mM NaCl. The purity of proteins was analysed by SDS-PAGE.

The DNAs encoding rN-MDP1 (residues 1–100) and rC-MDP1 (residues 101–209) were amplified from the full-length MDP1 gene that we had previously cloned[23]. The primers used for amplification of rN-MDP1 and rC-MDP1 were forward, 5'-gggtccttctgccgggagacgctgc-3', and reverse, 5'-caccaccaccaccaccactgagatcc-3', and forward,5'-gctgttaagcgtggtgtgggggccagtgca-3', and reverse,5'-cccaaccctccgaaaccagtggtcctcgtt-3', respectively. The amplified DNAs were ligated into a pET21b vector after cutting with Nde1 and Hind III. The integrity of the construct was confirmed by DNA sequencing. *E*. *coli* BL21 (DE3) pLysS (L1191) cells were transformed with the rN-MDP1–pET21b or rC-MDP1–pET21b construct. Similar to the protocol used for expressing rFull-MDP1, 40 mL of a small initial culture were inoculated into 4 L of fresh LB media, and IPTG was added approximately 2 h later to a final concentration of 0.5 μM. The culture was then cooled to 18 °C and further incubated for 16–18 h at 18 °C to express rN-MDP1. Similar to the method used for rFull-MDP1 preparation, the bacteria were washed with ice-cold PBS and suspended in buffer A at the same ratio. The bacteria were subsequently disrupted with an ultrasonic generator, and the resulting supernatant was loaded onto a His Trap column after removing aggregates by centrifugation and filtration as described above. The rN-MDP1 and rC-MDP1 were eluted with buffer A containing 500 mM imidazole. The extracted proteins were then precipitated with 80% saturated ammonium sulfate, similar to the protocol used for precipitating rFull-MDP1, and dialyzed at 4 °C in a buffer containing 50 mM sodium phosphate (pH 7.4) and 500 mM sodium chloride. The sample was then loaded onto an SP Sepharose (SP FF 5 mL 17515701, GE Healthcare) column and eluted with a linear gradient of 150–1,000 mM NaCl. The purity was examined by SDS-PAGE.

### Peptide mass mapping by TripleTOF MS/MS

Purified recombinant protein (rN-MDP1, 5 μg) was denatured in 30 μL of 2% SDS, 62.5 mM Tris-HCl (pH 6.8), 10% glycerol, and 2% 2-mercaptoethanol by heating for 10 min at 95 °C. The denatured protein was co-polymerized with 10% (T) polyacrylamide in a microcentrifuge tube[24]. The resulting gel was fixed in 50% methanol and 7% acetic acid, then reduced and carbamoidomethylated by 10 mM DTT and 50 mM iodeacetamide, respectively, and finally subjected to a conventional in-gel trypsin digestion. The peptide extract was dried in a vacuum centrifuge and subsequently dissolved in 0.2% trifluoroacetic acid (TFA) and 5% acetonitrile. The resulting peptide solution was processed sequentially through GL-Tip SDB and GL-Tip GC spin columns (GL Sciences, Tokyo, Japan) according to the manufacturer’s instructions. The purified peptide preparation was finally dissolved in 0.2% TFA and injected into a nano-flow LC (Eksigent expert 400, AB Sciex) coupled with a tandem mass spectrometer (TripleTOF5600+, AB Sciex). Analyses were conducted in duplicate under direct injection mode using a 75 μm × 15 cm, 3 μm ChromeXP C18 Chip column. Mobile phases A and B were 0.1% formic acid and 0.1% formic acid in acetonitrile, respectively. Peptides were eluted by using a 20-min gradient from 2% to 32% B at 300 nL/min. MS spectra (250 msec) followed by 10 MS/MS spectra (100 msec each) were acquired under the data-dependent mode.

Protein identification was carried out by using Mascot, version 2.2.1, (Matrix Science, London, UK) as a search engine with an in-house database generated from the NCBInr protein sequence database under the taxonomy of *M*. *tuberculosis* complex (18 July 2013 release). Modification settings were: fixed modification, carbamoidmethylation on cysteine; variable modifications, deamidated on asparagine and/or glutamine, N-terminal glutamine to pyroglutamate, N-terminal glutamate to pyroglutamate, and oxidation on methionine. A maximum of two missed cleavages was allowed. The significance threshold was set at 0.05 to give a false discovery rate of less than 5%. Only the proteins matched by two or more peptides with a score exceeding the “identity threshold” were reported.

### CD spectrum analysis

The protein was dialyzed in 50 mM sodium phosphate buffer (pH 7.0) containing 150 mM, 300 mM, 500 mM, 750 mM, 1000 mM, 1500 mM, or 2000 mM NaCl and pH titration experiments in steps of 1.0 (pH ranging from 7.5 to 0.5) adjusted to a final concentration of 3.2 μM. The CD spectra were measured at 25 °C in cells that were 1-mm in width as previously described[25, 26]. The CD spectra were recorded with a Jasco J-720.

### Sedimentation velocity (SV) analysis

The proteins were dialyzed against phosphate buffer (pH 7.0) containing 150 mM, 300 mM, or 500 mM NaCl, and the buffer (pH 0.5) containing 150 mM NaCl prior to performing a run. The SV experiments were performed at 20 °C with an Optima XL-I (Beckman Coulter) using an An50Ti rotor. Concentration gradients were measured by UV absorption at 230 nm without a time interval. The partial specific volume of the protein, buffer density, and viscosity were calculated by Sednterp[27]. The distribution functions of the sedimentation coefficients, c(s), were calculated by using the SEDFIT program, assuming that the frictional ratio was common to all the molecular species. The c(s) was converted to the distribution of the molecular weights, c(M), based on the Svedberg equation, which was implemented in SEDFIT[28].

### Glutaraldehyde crosslinking

The recombinant proteins rFull-MDP1 and rN-MDP1 were separately incubated in a 150 mM, 300 mM, 500 mM, 750 mM, 1000 mM, 1500 mM, or 2000 mM NaCl solution at room temperature for 30 min and were crosslinked by the addition of glutaraldehyde to a final concentration of 0.2% or were left untreated. The samples were then fractionated by SDS-PAGE with a 15% polyacrylamide gel, and the proteins were visualized by coomassie blue staining or silver staining.

### Study populations

The enrolled individuals are described in Table 1. The healthy control (HC) group consisted of 12 students (aged 20–24 years, males/females = 5/5) at Osaka City University Medical School (Osaka, Japan). They were negative for TB based on results from a chest x-ray and immune-based assessments (tuberculin skin tests and IGRA, QuantiFERON TB-2G (QFT) (Cellestis, Valencia, CA, USA), and they were not suspected of having any risks of *M*. *tuberculosis* infection, such as HIV infection, close contact with active TB-infected individuals, or chest x-ray findings. The active TB group consisted of eight individuals (aged 23–74 years, M/F = 6/2) diagnosed with active TB based on microbiologic examinations using either a positive culture for *M*. *tuberculosis* or a positive DNA amplification test specific for *M*. *tuberculosis* (TRC Test; TRCRapid-160, Tosoh, Tokyo, Japan) from sputum specimens. A positive QFT was obtained for all cases in this group. The past TB group consisted of 12 plus 23 patients who had a definitive past history of pulmonary TB more than 5 years previously. Their bacteriologic examinations were negative in the sputum culture and nucleic acid amplification *M*. *tuberculosis* tests. Their chest x-rays each showed sclerotic lesions and stable cavities. Because no infiltrating shadows were found around these cavities, the cavitary lesions indicated a radiographic diagnosis of TB. In this group, 33% of individuals were QFT-positive. Subjects were excluded from this study when disease due to nontuberculous mycobacteria (NTM) was confirmed by repeated cultures and satisfied the American Thoracic Society guidelines [29]. The serum specimens were assayed without knowledge of the patients’ clinical characteristics. The studies conducted using human subjects were approved by the research and ethical committees of the National Toneyama Hospital (2009–0920) and Osaka City University Graduate School of Medicine (1458), and informed consent was obtained from all subjects by written and approved by the committees. All methods employed in this study were performed in accordance with the relevant guidelines and regulations.

**Table 1:** Characteristics of the study population.

	Healthy control (HC)	Active TB	Past TB-1	Past TB-2
Number of participants	10	8	12	23
Age, mean (years)±SD	21.1±1.14	44.13±17.28	72.33±10.59	68.00±11.11
Age range (years)	20–24	23–74	57–85	51–89
Male/female ratio	5/5	6/2	4/8	10/13
IGRA positive (%)	0	100	33.33	60.9

IGRA: interferon-gamma release assay; SD, standard deviation; TB, tuberculosis

### Enzyme-linked immunosorbent assay

Ninety-six-well microplates (Sumitomo) were coated with rFull-MDP1 (0.1 μg/ml), rN-MDP1 (0.1 μg/ml), CFP10 (0.5 μg/ml), EAST-6 (0.5 μg/ml), Antigen 85B (Ag85) (0.5 μg/ml), or purified protein derivatives (PPD) (0.5 μg/ml) by overnight incubation at 4 °C. The plates were then blocked with PBS containing 5% skim milk and 0.05% Tween 20 overnight at 4 °C. The wells were washed four times with PBS containing 0.05% Tween 20 (PBS-T). Human serum samples diluted 1:200 by PBS-T and 0.5% skim milk were added to the wells and incubated for 1 h at 37 °C. After washing the wells four times with 300 μl of PBS-T, horse radish peroxidase-conjugated anti-human IgG antibody was added. After incubation at 37 °C for 1 h, the plates were washed four times with PBS-T. Colour development was performed by the addition of SureBlue/TMB peroxidase substrate (Sera/Care Life Science Company, Gaithersburg, MD, USA) for around 10 min and was stopped by the addition of 20 μL of 6 M HCl. The optical density of the sample was measured at 450 nm.

### Statistical analyses

Optical density differences between study groups were determined using box-and-whisker plot. ROC curve analysis and the AUC for each antigen were calculated with IBM SPSS software Ver. 21 and 22 (Armonk, New York, USA).

## Results

### Structure prediction of MDP1

**Fig 1:**
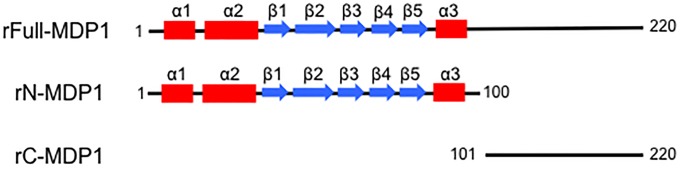
Schematic of the MDP1 secondary structure prediction by GTOP. GTOP was used to predict the secondary structure of MDP1, and schematics of the findings are shown. The N-terminal half of MDP1 contains three alpha helix portions (alpha 1–3; red boxes) and five beta sheet regions (beta 1–5; blue arrows). In contrast, the C-terminal half of MDP1 does not show a stable structure, suggesting the presence of intrinsically disordered regions (IDRs). Each number indicates the amino acid position in MDP1.

We performed a structure prediction for *M*. *tuberculosis* MDP1 (Rv2986c) by using GTOP software (National Institute of Genetics Japan). The results revealed that MDP1 contains two distinct domains, as shown in Fig 1. The N-terminal domain shares characteristics with the bacterial histone-like protein HU. It was recently reported that this region forms dimers with long symmetric arms, which are presumably responsible for DNA-binding[19]. In contrast, the C-terminal domain, including six PAKK repetitive sequences, did not show any secondary structure, suggesting that the C-terminal half has IDRs (Fig 1).

### Purification of recombinant MDP1 proteins

We expressed amino acids 1–100 of MDP1 as a histidine-tagged protein (rN-MDP1) in *Escherichia coli* BL21 (DE3) pLysS. We purified rN-MDP1 from the lysates by first using a nickel affinity chromatograph (Part A of S2 Fig). We then repeatedly precipitated MDP1-rich fractions with an ammonium sulphate precipitation to remove contaminating nucleic acids, which was not involved in the method that Bhowmick et al used [19]. We then performed heparin column chromatography and finally cation exchange column chromatography (Part B of S2 Fig). We confirmed that the purified recombinant protein was rN-MDP1 via mass spectrometric identification (S1 Fig) and western blotting using a specific monoclonal antibody that recognizes the amino acids 51–70 region of MDP1 (mab7C)[7]. As shown in Part B of S2 Fig, single bands of rN-MDP1 were observed for fractions 19 to 21 (150 mM to 1,000 mM NaCl gradients) in the final ion exchange column chromatography, and their absorbance levels at 260 nm were each below 0.05 per 1 mg protein, indicating that contaminating DNA was removed from the purified protein.

We then purified full-length HIS-tagged recombinant MDP1 (rFull-MDP1). Similar to the method used for purification of rN-MDP1, we first applied the lysate of bacteria that expressed rFull-MDP1 to nickel column chromatography (Part A of S3 Fig). The rFull-MDP1-rich fraction was then applied to heparin column chromatography and eluted by a NaCl gradient. The rFull-MDP1-rich fractions were then precipitated with an ammonium sulphate precipitation and further fractionated with cation exchange chromatography (CM sepharose) with a density gradient of NaCl. The resulting eluates were analysed by SDS-PAGE and are shown in Part B of S3 Fig. We examined the contamination of nucleic acids in fractions 4 to 8 and found that the absorbance at 260 nm is below 0.01 per 1 mg protein, indicating that the purification procedure successfully removed contaminating nucleic acids.

### Analysis of MDP1 secondary structures

To obtain experimental evidence for the secondary structure prediction of the recombinant proteins, we performed analyses using circular dichroism (CD) spectroscopy. These analyses showed that the alpha-helix content of rFull-MDP1 purified by the present protocol was 20%, whereas the corresponding protein purified by acid extraction showed a 1.4% value, indicating that acid extraction really disrupts the protein structure (S4 Fig). Similarly, the alpha-helix content of rN-MDP1, purified by the present protocol or by acid extraction was 35% or 9.7%, respectively, supporting the finding that the previous protocol denatured the MDP1 protein.

**Fig 2:**
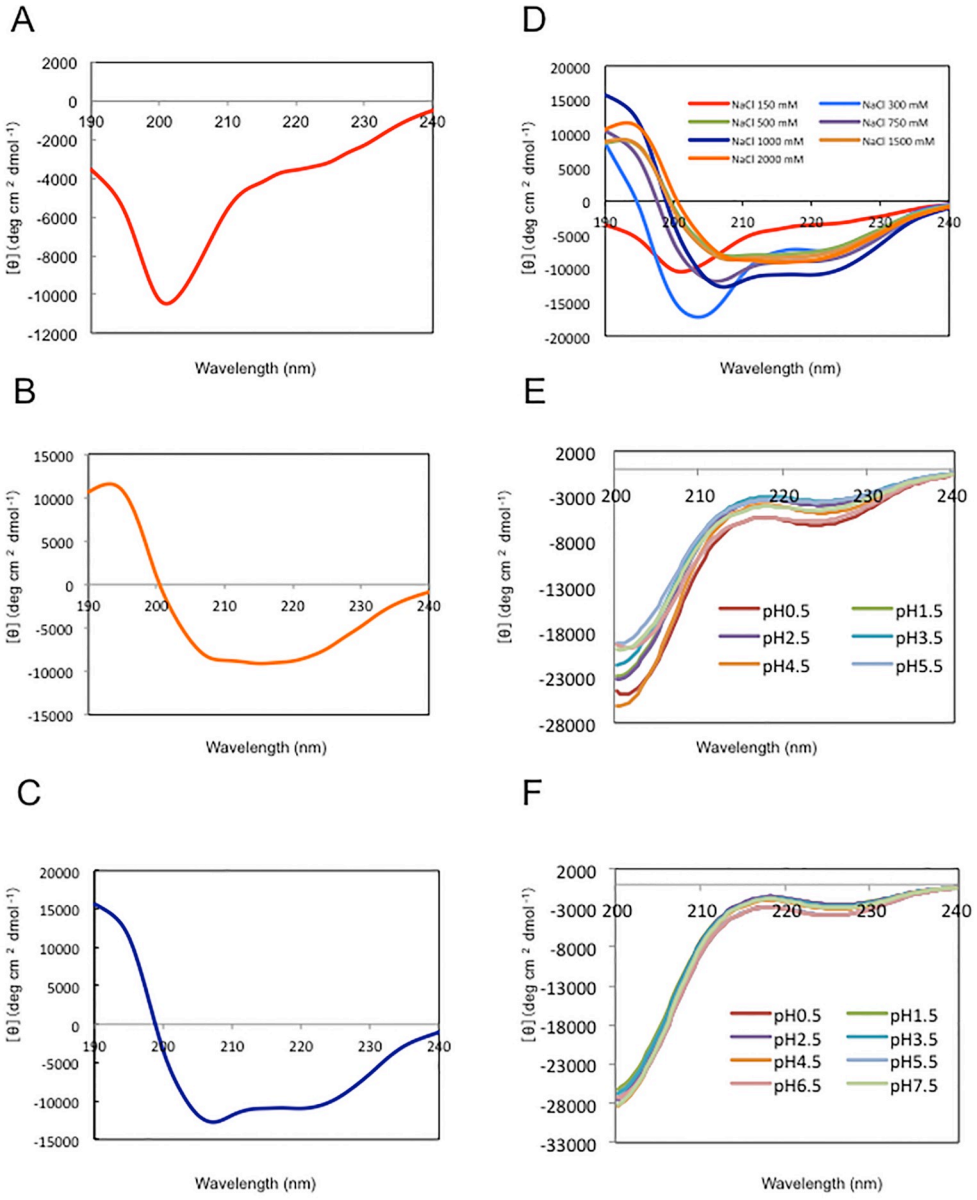
CD spectroscopy measurements of rN-MDP1 and rFull-MDP1. The secondary structural changes of rN-MDP1 and rFull-MDP1 with different salt concentrations were monitored by CD spectroscopy studies. (A–C) CD spectra of rN-MDP1 in phosphate buffer (pH 7.0) containing (A) 150 mM NaCl, (B) 1,000 mM NaCl, or (C) 2,000 mM NaCl. (D) Merged CD spectra of rN-MDP1 in phosphate buffer pH 7.0 containing 150 mM, 300 mM, 500 mM, 750 mM, 1,000 mM, 1,500 mM, or 2,000 mM NaCl. Merged CD spectra of rN-MDP1 (E) and rFull-MDP1 (F) at pH0.5, 1.5, 2.5, 3.5, 4.5, 5.5, 6.5 and 7.5 in buffer (pH 7.0) containing 150 mM NaCl.

We also analysed the effects of salt concentration on the secondary structure of rN-MDP1 purified by the present control. The recombinant proteins were dialyzed using buffers with various NaCl concentrations ranging from 150 mM to 2,000 mM, and then their CD spectra were measured. The resulting data revealed that the alpha-helix contents gradually increased and approached the predicted values as the salt concentrations increased, as shown in Fig 2A–2C. This indicates that higher salt concentrations stabilize the structure of the N-terminal half of MDP1 (Fig 2D).

Recently it was shown that *Helicobacter pylori* HU is likely to be stabilized under acidic conditions [30], which is structural and sequence homolog of N-terminal MDP1. We performed pH titration experiments in steps of 1.0 (pH ranging from 0.5 to 7.5) to know the acid denaturation behavior of rN-MDP1 (Fig 2E) and rFull-MDP1 (Fig 2F) in the physical concentration of NaCl (150 mM). As shown in Fig 2E and 2F, there is no obvious change of CD spectra depending on pH range in both rN-MDP1 and rFull-MDP1. This suggests different characteristics of HU proteins in *M*. *tuberculosis* and *H*. *pylori*.

### Analysis of oligomerization and influence of salt concentration

**Fig 3:**
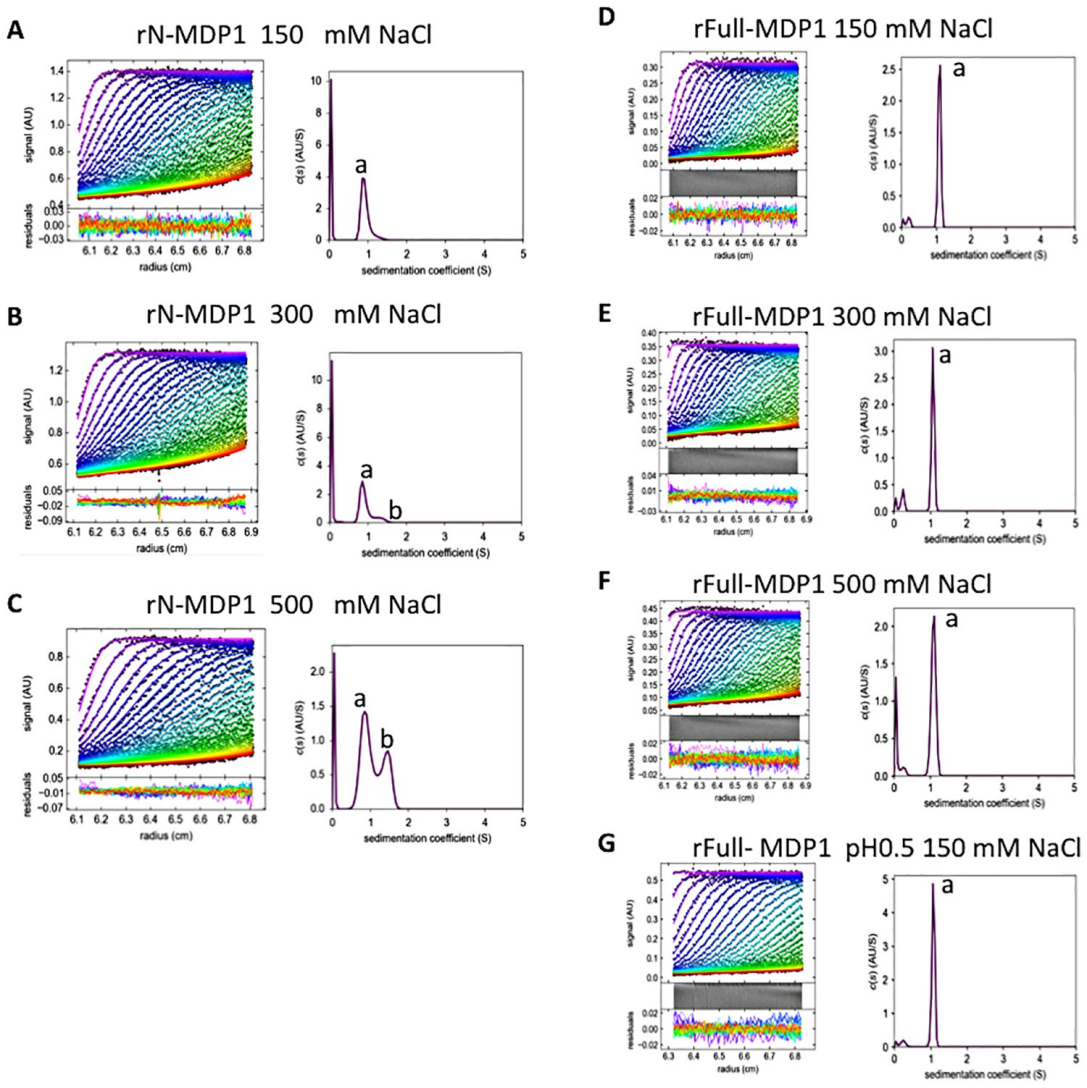
Sedimentation velocity measurements. Left: Radial fluorescence scans (dot colours indicate times in the following order purple-blue-green-yellow-red) during sedimentation at 20 °C. Solid lines are the best-fit with a single species. Right: The resulting sedimentation coefficient distributions. The peak of “a” and “b” was estimated to be the monomer and dimer, respectively. (A–G). Results for rN-MDP1 in buffer containing 150 mM (A), 300 mM (B), or 500 mM (C) NaCl and those for r-Full-MDP 1 in buffer containing 150 mM (D), 300 mM (E) 500 mM (F). r-Full-MDP 1 in 150 mM NaCl buffer pH 0.5 (G).

We next studied the oligomerization state of MDP1. We dialyzed rN-MDP1 and rFull-MDP1 against Tris-HCl buffer (pH 7.0) containing 150 mM, 300 mM, or 500 mM NaCl and subjected the resulting products to sedimentation velocity (SV). Analyses of the SV data with SEDFIT software indicated that rN-MDP1 is a monomer when the salt concentration is 150 mM (Fig 3A), but higher salt concentrations induced dimerization of rN-MDP1 at rates of 17% at 300 mM (Fig 3B) and 34% at 500 mM (Fig 3C). In contrast, rFull-MDP1 was monomer in every salt concentration we tested (Fig 3D–3F). These suggested that C-terminal IDR region disturbs dimerization of MDP1.

Because SV measurement cannot be applied for molecules in high salt buffer, we performed glutaraldehyde crosslinking assays to determine the oligomerization status of MDP1. We incubated rN-MDP1 in solvent containing 150 mM, 300 mM, 500 mM, 750 mM, 1,000 mM, 1,500 mM, or 2,000 mM NaCl in the presence or absence of 0.2% glutaraldehyde, and the resulting products were subjected to SDS-PAGE. As shown in Part A of S5 Fig, the amount of rN-MDP1 monomer was decreased as the salt concentration increased, and most rN-MDP1 formed dimers at 1,500 mM and 2,000 mM NaCl.

We also analysed the oligomerization of rFull-MDP1 in Tris buffer containing 150 mM, 300 mM, 500 mM, 1,000 mM, 1,500 mM, or 2,000 mM NaCl. We could not find any obvious dimers in 150 mM, 300 mM, or 500 mM, NaCl as analyzed SV measurement. In contrast, dimers clearly appeared at 1,000 mM NaCl, and most of the recombinant proteins dimerized at 2,000 mM NaCl (Part B of S5 Fig). Taken together, these data demonstrate that MDP1 forms dimers in solvents with high salt concentrations.

### Evaluation of the antigenicities of recombinant MDP1 to IgG from *M*. *tuberculosis*-infected individuals

We then tested the antigenicities of the recombinant MDP1 proteins that were purified in this study and compared them with those of the corresponding recombinant proteins purified by the previous method[15, 16]. We used recombinant CFP10, ESAT6, and Ag85, and PPD as a control[15]. The CFP10 and ESAT6 proteins, produced from *M*. *tuberculosis* during the growth phase[31], is used in IGRA diagnostic tests for the detection of *M*. *tuberculosis* infection[32]. Ag85 is a major secretary protein and its expression is limited at early phase of *M*. *tuberculosis* infection [33, 34]. We collected blood from eight active tuberculosis patients and 12 past tuberculosis individuals, as shown in Table 1. We also collected blood from 10 healthy individuals with IGRA-negative results and normal chest x-rays.

**Fig 4:**
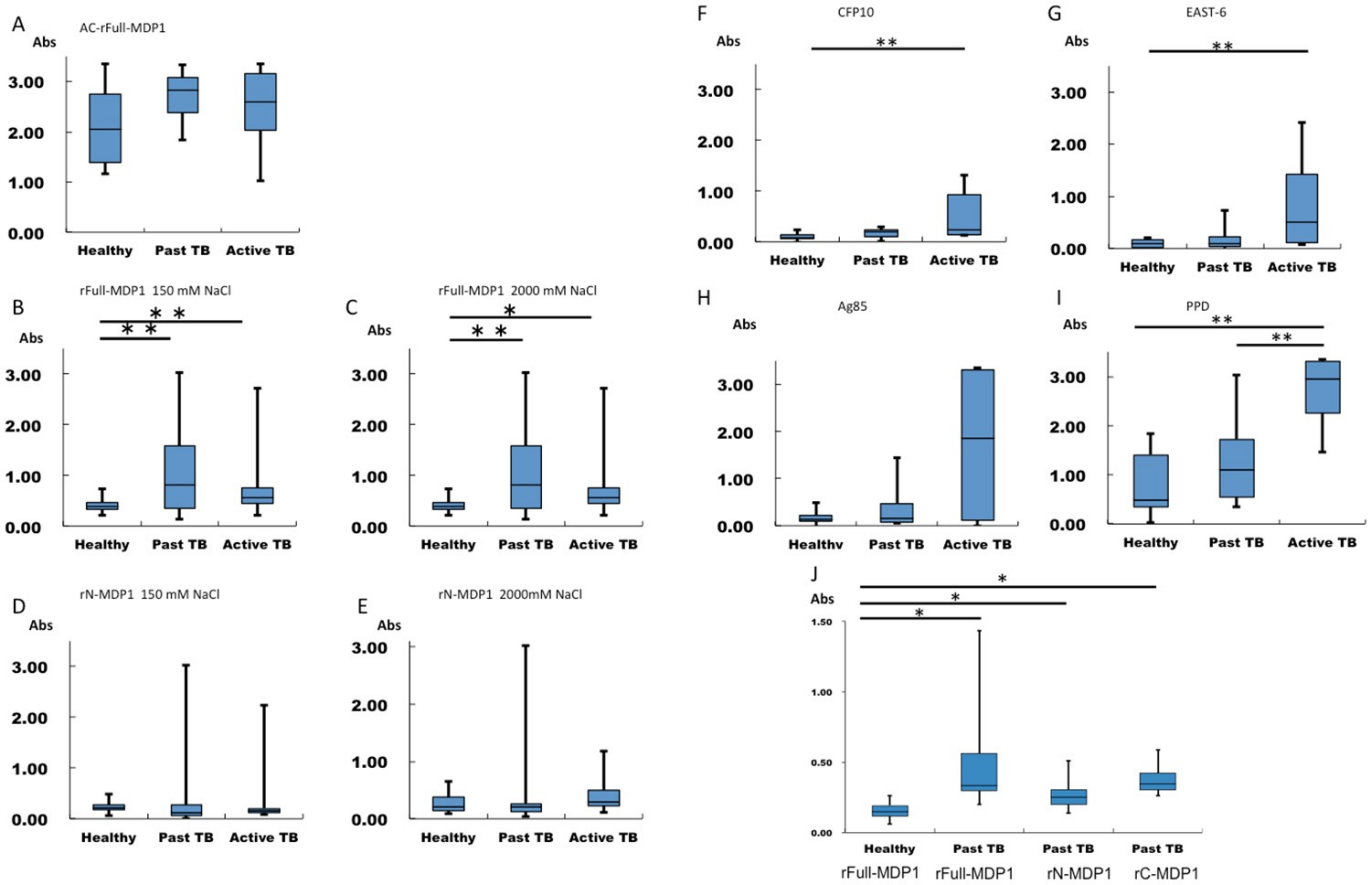
ELISAs to detect human MDP1, CFP10, ESTA6, Ag85, and PPD-specific IgG antibodies. The results of ELISAs performed to detect IgG antibodies that recognize recombinant proteins and PPD in human blood samples. The tested sera in A-I were from 12 past tuberculosis (Past TB) patients, 8 active tuberculosis patients (Active TB), and 10 healthy control (Healthy) individuals (also for J). Additionally, sera derived from 23 individuals with past tuberculosis were tested in Fig. 4J. (A) IgG responses to AC-rFull-MDP1, which was purified by acid extraction. (B–C) IgG responses to rFull-MDP1, purified by the refined purification method, was immobilized in buffer containing 150 mM NaCl (B) or 2 M NaCl (C). (D–E) IgG responses to rN-MDP1 immobilized in 150 mM NaCl (D) and 2 M NaCl (E). (F–I) IgG responses to CFP10 (F), ESAT6 (G), Ag85 (H), and PPD (I). (J) IgG responses to rFull-MDP1 of the 10 healthy control and those to rFull-MDP1, rN-MDP1, and rC-MDP1 of 23 other past TB individuals. *, p<0.05. **p<0.01.

We first coated plates with CFP10, ESAT6, Ag85, PPD, and rFull-MDP1, and detected antigen-specific IgG in the blood samples by ELISA. The results revealed that the IgG responses to CFP10, ESAT6, Ag85, and PPD are significantly higher in active tuberculosis patients (Fig 4F–4I), implying its merit for the detection of active tuberculosis. When we used AC-rfull-MDP1, the average of IgG level trended upward in the following order: healthy control, active tuberculosis, and past tuberculosis; however, the differences between these groups are not statistically significant (Fig 4A). In contrast, when we used the equivalent protein purified by the current method, the background IgG reaction from healthy controls was decreased and the anti-MDP1 IgG level was significantly higher in past tuberculosis patients than in any other group (Fig 4B).

The oligomerization analysis results suggest that salt concentration affects the secondary structure and polymerization of MDP1 (Part A and Part B of S5 Fig). Therefore, we examined the effects of salt concentration on the detection of MDP1-specific IgG. We dialyzed rFull-MDP1 against a solution containing 150 mM (monomer) or 2 M NaCl (dimer) and used the results to coat ELISA plates. The resulting IgG ELISA data showed that both the monomeric and dimeric rFull-MDP1 have the similar antigenicity, suggesting that dimerization is not important for recognition by IgG in the tested samples (Fig 4B and 4C).

We performed receiver operating characteristic (ROC) analyses and presented the data in Table 2. Area under the curves (AUCs) of rFull-MDP1 immobilized in 150 and 2,000 mM NaCl were 0.817 (95% CI 0.637–0.996, *p* = 0.012) and 0.792 (95% CI 0.415–0.902, *p* = 0.021) in analysis between the past tuberculosis and HC groups. In contrast, those of CFP10, ESAT6, Ag85, and PPD were 0.633 (95% CI 0.388–0.879, *p* = 0.291), 0.575 (95% CI 0.33–0.82, *p* = 553), 0.558 (95% CI 0.308–0.809, *p* = 0.644), and 0.700 (95% CI 0.472–0.928, *p* = 0.114), respectively. These data show that rFull-MDP1 purified by the current method is useful for detecting asymptomatic tuberculosis that cannot be detected by IgG against CFP10, ESAT6, and Ag85.

**Table 2:** ROC analysis.

Antigens	Healthy vs Past TB			Healthy vs Active TB			Active TB vs Past TB		
	AUC	95% CI	*p*-value	AUC	95% CI	*p*-value	AUC	95% CI	*p*-value
MDP1 (150 mM)	0.817	0.637–0.996	0.012*	0.775	0.55–1	0.05*	0.625	0.367–0.883	0.335
MDP1 (2M)	0.792	0.415–0.902	0.021*	0.788	0.556–1	0.041*	0.677	0.427–0.927	0.19
CFP10	0.633	0.388–0.879	0.291	0.85	0.667–1	0.013*	0.333	0.062–0.604	0.217
ESAT6	0.575	0.33–0.82	0.553	0.825	0.63–1	0.037*	0.219	0.01–0.428	0.099
Ag85	0.558	0.308–0.809	0.644	0.563	0.272–0.853	0.657	0.323	0.025–0.621	0.323
PPD	0.7	0.472–0.928	0.114	0.85	0.673–1	0.013*	0.115	0–0.258	0.004*

MDP1 (150 mM), rFull-MDP1 was immobilized in the buffer containing 150 mM NaCl. MDP1 (2 M), rFull-MDP1 was immobilized in the buffer containing 2 M NaCl.

* *p*<0.05.

We lastly addressed whether or not the C-terminal IDRs affect the recognition of MDP1 by IgG (Fig 4B–4E). We compared the IgG binding levels between rN-MDP1 and rFull-MDP1 and found that the antigenicity of rN-MDP1 is remarkably lower than that of rFull-MDP1, suggesting importance of C-terminal IDRs in the recognition by human IgG (between Fig 4B and 4E, or 4C and 4E).

In order to study whether C-terminal IDR is substantially recognized by human IgG, we expressed the C-terminal half IDR domain of MDP1 (rC-MDP1) consisting 101–209 amino acids of MDP1, as histidine-tagged protein and purified by the nickel column chromatograph. We assessed its antigenicity by IgG-ELISA by using another set of sera derived from 23 individuals with past tuberculosis (Table 1). The data showed that IgG response is higher to rC-MDP1 than rN-MDP1 but was lower than rFull-MDP1 (Fig 4J). This suggest the predominant recognition of C-terminal IDRs by MDP1-specific IgG and importance of whole protein structure in the IgG recognition.

## Discussion

MDP1 has growth-arresting activity and is predicted to be a major antigen of persistent *M*. *tuberculosis*. As such, it is also presumed to be a marker of asymptomatic tuberculosis. In this study, we established a new method to purify recombinant MDP1, which adopted the predicted structure after this purification, and showed the usefulness of this recombinant protein in the development of a diagnostic test for asymptomatic tuberculosis.

The structure prediction of MDP1 by GTOP (Fig 1) suggested that the C-terminal half of MDP1 is intrinsically disordered. Interest in IDPs has recently been increasing because IDPs have a large variety of important cellular functions [35, 36]. IDPs are ubiquitous in eukaryotes, and long IDRs occupy 33% of the entirety of eukaryotic proteins[20]. Eukaryotic histones are the most well-known examples of IDPs that contain long IDRs. Most notably, histone tails, corresponding to IDRs, play important roles in chromatin functions related to epigenetics[35–37]. In contrast to eukaryotes, long IDPs are rare in bacteria or archaea, as revealed from estimated contents of only 2% and 4% in archaeal and eubacterial proteins, respectively[20]. In fact, most histone-like proteins in bacteria do not contain IDRs, except for MDP1 homologues. Notably, the C-terminal IDRs of MDP1 have DNA-binding activities [38–40], implying its involvement in the construction of nucleoid architecture. Indeed, our current study showed the important role of C-terminal IDR in MDP1-functions [41].

Acid-extracted MDP1 was previously eluted in the 195-kDa fraction of a gel filtration chromatograph[10]. Additionally, the x-ray crystal structure of rN-MDP1 indicated that the protein forms a dimeric structure [19]. In this study, the SV data analysed by SEDFIT indicated that 97% of the protein is monomeric, which suggests that the gel filtration analysis in the previous study overestimated the molecular weight of MDP1[10]. This discrepancy could be related to the aggregation of MDP1 protein, which is denatured by acid extraction (S4 Fig). Here, the results of SV measurements and glutaraldehyde crosslinking assays demonstrate that both the rFull-MDP1 and rN-MDP1 were monomeric at the physiological salt concentration (Fig 3 and S5 Fig).

Notably, our results also revealed that both the rFull-MDP1 and rN-MDP1 form dimers as the salt concentration increases (Fig 3 and S5 Fig). The CD analysis showed that alpha-helical contents of rN-MDP1 increase in progressively higher salt concentrations. This implies that the dimer formation is coupled with content of secondary structures. It was reported that most other bacterial HU-like proteins form dimers at 150 mM NaCl^33^. In this context, the MDP1 dimer appears to be more unstable than these other related proteins. We presume that rN-MDP1 could be crystallized as a dimer because of a high salt concentration, such as 3 M sodium formate [19]. Furthermore, it is conceivable that MDP1 may be interchangeable between monomeric and dimeric states in the crowded cytoplasm of mycobacteria [42].

ELISA experiments revealed that rFull-MDP1, purified by the present method, may be useful for the diagnosis of asymptomatic tuberculosis (Fig 4B and 4C). This highlights the importance of preserving native protein structures in serodiagnostic assays. The results of our IgG binding level comparison between rFull-MDP1 and rN-MDP1 or rC-MDP1 suggest that IDRs are functional in the recognition of MDP1 by human IgG (Fig 4B–4E). There are two types of epitopes recognized by antibodies. A “continuous epitope” is a part of the amino acid sequence of a protein, while a “discontinuous epitope” consists of residues from different parts of the protein sequence. It was reported that discontinuous epitopes are more frequent than continuous ones[43]. The precise prediction of antibody epitopes is not yet possible, but surface accessible regions[44], flexible portions[45], and portions protruding from the protein’s globular surfaces[46] are correlated with antibody epitopes[47]. Based on this consideration, the IDRs of MDP1 may be feasible targets of IgG because IDRs possess several of these characteristics; they are surface exposed, flexible, and protruded. Nevertheless, IgG titer to rC-MDP1 was lower than rfull-MDP1 (Fig 4J), suggesting the demand for entire polypeptide structure in recognition of MDP1-IDR by human IgG.

The level of IgG antibodies against CFP10, ESAT6, Ag85, and PPD was elevated in patients with active tuberculosis (Fig 4F–4I). CFP10 forms a heterodimer with ESAT6, which has pore-forming activity in the phagosomal membrane[48, 49]. This activity of the CFP10–ESAT6 complex is involved in the virulence of *M*. *tuberculosis* and in active disease[50]. Ag85 is a mycolyltransferase involved in the final stages of mycobacterial cell wall assembly [51] and its expression is limited at early phase of *M*. *tuberculosis* infection [33, 34]. PPD is heat-inactivated culture filtrate of *M*. *tuberculosis* at early growth stage in *in vitro*. Accordingly, our data show that the immune response to CFP10, ESAT6, Ag85 and PPD are higher while the immune response against MDP1 is lower in patients with active tuberculosis than in patients with past tuberculosis. This implies that an evaluation of the ratio of immune responses to CFP10–ESAT6 and Ag85 vs MDP1 might be useful for determining the disease status of tuberculosis.

Currently, IGRAs are clinically applied for the detection of asymptomatic tuberculosis[32, 52]. However IGRA-negative subjects sometimes develop tuberculosis and IGRAs are also expensive and have complicated handling as a point-of-care test [3, 4, 52, 53]. The serodiagnostic kits that have been developed thus far are not recommended for the detection of active tuberculosis. However, serodiagnosis has the benefit of predicting disease progression because antibody levels are correlated with the amounts of antigens that typically increase before disease progression[54]. It also has the advantage of reducing the cost of diagnostic kits by replacing with present cell culture-based diagnostic methods, such as IGRA, and allowing easy handling.

Multiple antigens produced by persistent *M*. *tuberculosis* may be useful for the detection of IGRA-negative asymptomatic infections. Although further evaluation of a combination of biomarkers is necessary, anti-MDP1 antibody may be a potential marker of asymptomatic tuberculosis. Besides, several recent reports have shown the importance of antibodies in host protection against tuberculosis [16, 55–58]. Higher level of protective IgGs were produced more in the individuals with LTBI than active tuberculosis patients [57]. There is the possibility that MDP1-antibodies contribute to host protection in asymptomatic status. This is partially supported by our current study of the *M*. *tuberculosis*-infected individuals [16] and MDP1 vaccination indeed induced the protection in mice against tuberculosis [59]. Taking the tertiary structures of antigens into consideration will be important for both of developments of serodiagnosis and vaccines for tuberculosis.

## Supporting information

S1 Fig A mass spectrometric analysis of purified rN-MDP1 is shown. Sequence coverage was 84% in the N-terminal 100 amino acid sequence of MDP1; these amino acids are marked in red.
(TIFF)

S2 Fig (A) A representative gel resulting from an SDS-PAGE analysis of the proteins fractionated by a HIS-trap column. Recombinant *E*. *coli* expressing rN-MDP1 were lysed by sonication and centrifuged. The supernatant was then loaded onto a His-Trap column in the presence of 10 mM imidazole and eluted by 500 mM imidazole. Lane 1: lysates after disruption of the bacteria; lane 2: applied supernatants of bacterial lysates; lane 3: column flow-through; lanes 4–7: fractions 7–10, respectively; and M, molecular weight marker. (B) A representative gel resulting from an SDS-PAGE analysis of the proteins fractionated by ion exchange column chromatography. The proteins were passed thorough an ion exchange column and eluted with a linear gradient of 150–1,000 mM NaCl. Lane 1: applied sample; lane 2: column flow-through; lanes 3–10: fractions 16–23, respectively; and M, molecular weight marker. Original gel images of S2-A and S2-B are shown in S2-C and S2-D, respectively.
(TIFF)

S3 Fig (A) A representative gel resulting from an SDS-PAGE analysis of the proteins fractionated by a HIS-trap column. Recombinant *E*. *coli* expressing rFull-MDP1 were lysed by sonication and centrifuged. The supernatant was then loaded onto a His-Trap column in the presence of 10 mM imidazole and eluted by 300 mM imidazole. Lane 1: lysates after disruption of the bacteria; lane 2: applied supernatants of bacterial lysates; lane 3: column flow-through; lanes 4–11: fractions 16–23, respectively; and M, molecular weight marker. (B) A representative gel resulting from an SDS-PAGE analysis of the proteins fractionated by ion exchange column chromatography. The rFull-MDP1 purified by heparin column chromatography was further purified by CM Sepharose column chromatography. The proteins were eluted with a linear gradient of 100–1,000 mM NaCl. Lane 1: applied sample after heparin column purification; lane 2: column flow-through, lanes 3–8: fractions 14–19, respectively; and M, molecular weight marker. Original gel images of S3-A and S3-B are shown in S3-C and S3-D, respectively.
(TIFF)

S4 Fig A comparison between the secondary structures of rFull-MDP1 purified by the different methods based on CD spectroscopy studies. (A) CD spectra of rFull-MDP1 purified through acid extraction. (B) CD spectra of rFull-MDP1 purified by the refined method without acid extraction. Proteins were resolved in phosphate buffer (pH 7.0) containing 150 mM NaCl.
(TIFF)

S5 Fig SDS-PAGE analysis of rN-MDP1 (A) and rFull-MDP1 (B) with or without cross-linking by glutaraldehyde. The proteins were cross-linked at various concentrations of NaCl and fractionated with SDS-PAGE. The gels were stained with CBB (A) and silver staining (B). Original gel images of S5-A and S5-B are shown in S5-C and S5-D, respectively.
(TIFF)
