# Correction: Significance of a histone-like protein with its native structure for the diagnosis of asymptomatic tuberculosis

**DOI:** 10.1371/journal.pone.0256946

**Published:** 2021-08-27

**Authors:** Yukiko Ohara, Yuriko Ozeki, Yoshitaka Tateishi, Tsukasa Mashima, Fumio Arisaka, Yasuo Tsunaka, Yoshie Fujiwara, Akihito Nishiyama, Yutaka Yoshida, Kengo Kitadokoro, Haruka Kobayashi, Yukihiro Kaneko, Ichiro Nakagawa, Ryoji Maekura, Saburo Yamamoto, Masato Katahira, Sohkichi Matsumoto

In the Study populations subsection of the Materials and methods section, there is an error in the second sentence. The correct sentence is: The healthy control (HC) group consisted of 12 students (aged 20–24 years, males/females = 6/6) and 23 students (aged 20–28 years, males/females = 14/9) at Osaka City University Medical School (Osaka, Japan).

There are a number of errors in the caption for Fig 4, “ELISAs to detect human MDP1, CFP10, ESTA6, Ag85, and PPD-specific IgG antibodies.” Please see the complete, correct Fig 4 caption here.

**Fig 4:**
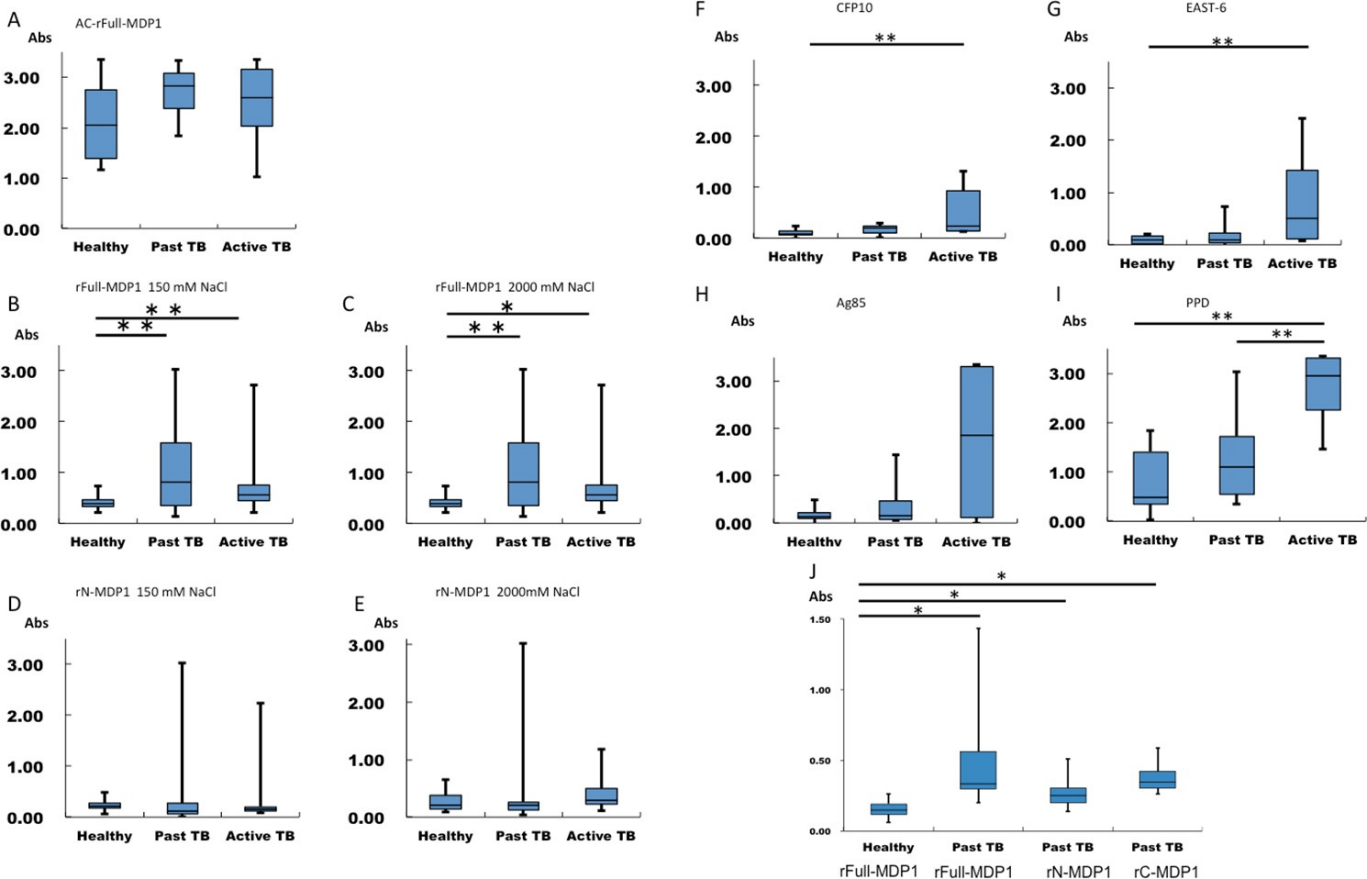
ELISAs to detect human MDP1, CFP10, ESTA6, Ag85, and PPD-specific IgG antibodies. The results of ELISAs performed to detect IgG antibodies that recognize recombinant proteins and PPD in human blood samples. The tested sera in A-I were from 12 past tuberculosis (Past TB) patients, 8 active tuberculosis patients (Active TB), and 12 healthy control (Healthy) individuals (also for J). Additionally, sera derived from 23 individuals with past tuberculosis were tested in Fig 4J. (A) IgG responses to AC-rFull-MDP1, which was purified by acid extraction. (B–C) IgG responses to rFull-MDP1, purified by the refined purification method, was immobilized in buffer containing 150 mM NaCl (B) or 2 M NaCl (C). (D–E) IgG responses to rN-MDP1 immobilized in 150 mM NaCl (D) and 2 M NaCl (E). (F–I) IgG responses to CFP10 (F), ESAT6 (G), Ag85 (H), and PPD (I). (J) IgG responses to rFull-MDP1 of the 10 healthy control and those to rFull-MDP1, rN-MDP1, and rC-MDP1 of 23 other past TB individuals.

In Table 1, the values in the healthy control group “Healthy control (HC)-2" are missing and should appear as the fourth column of the table. The number of participants under the column “Healthy control (HC)-1” should be 12 and the male/female ration under the column “Healthy control (HC)-1” should be 6/6. Please see the correct Table 1 here.

**Table 1:** Characteristics of the study population.

	Healthy control (HC)-1	Active TB	Past TB-1	Healthy control (HC)-2	Past TB-2
Number of participants	12	8	12	23	23
Age, mean (years)±SD	21.1±1.14	44.13±17.28	72.33±10.59	22.13±1.63	68.00±11.11
Age range (years)	20–24	23–74	57–85	20–28	51–89
Male/female ratio	6/6	6/2	4/8	14/9	10/13
IGRA positive (%)	0	100	33.33	0	60.9

IGRA: interferon-gamma release assay; SD, standard deviation; TB, tuberculosis

## References

[1] Ohara Y, Ozeki Y, Tateishi Y, Mashima T, Arisaka F, Tsunaka Y, et al. (2018) Significance of a histone-like protein with its native structure for the diagnosis of asymptomatic tuberculosis. PLoS ONE 13(10): e0204160. 10.1371/journal.pone.0204160 30359374 PMC6201868

