# Supplementary figures and images for: Significance of a histone-like protein with its native structure for the diagnosis of asymptomatic tuberculosis
Source: PLoS One. 2018 Oct 25;13(10):e0204160. doi: 10.1371/journal.pone.0204160 (PMC6201868; doi:10.1371/journal.pone.0204160)

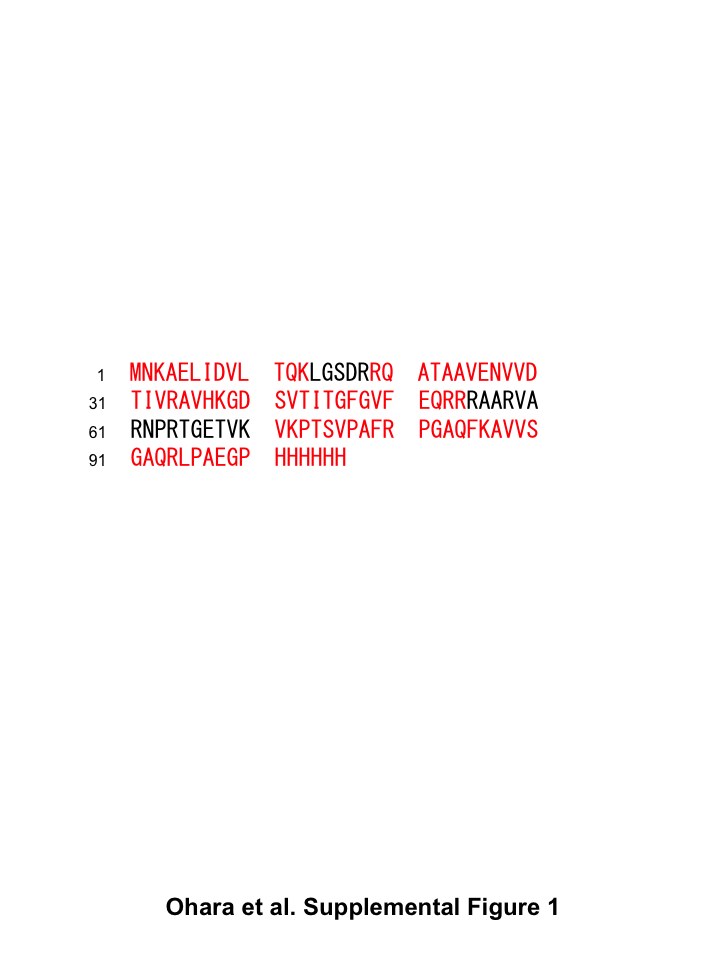

Supplement: S1 Fig — Sequence coverage was 84% in the N-terminal 100 amino acid sequence of MDP1; these amino acids are marked in red. (TIFF) [file pone.0204160.s001.tiff]

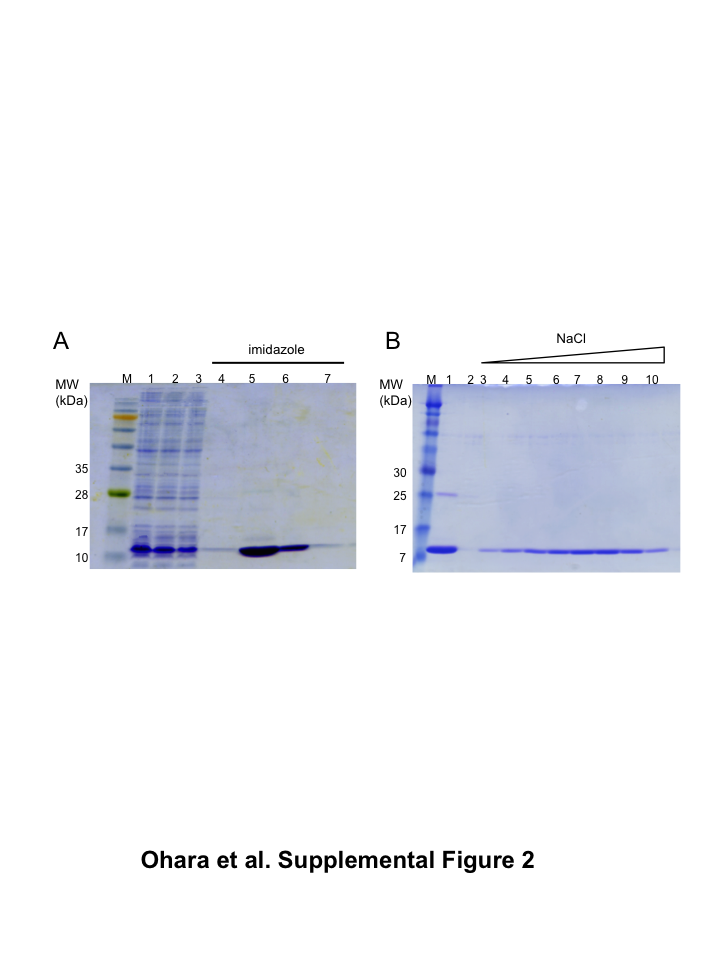

Supplement: S2 Fig — (A) A representative gel resulting from an SDS-PAGE analysis of the proteins fractionated by a HIS-trap column. Recombinant E. coli expressing rN-MDP1 were lysed by sonication and centrifuged. The supernatant was then loaded onto a His-Trap column in the presence of 10 mM imidazole and eluted by 500 mM imidazole. Lane 1: lysates after disruption of the bacteria; lane 2: applied supernatants of bacterial lysates; lane 3: column flow-through; lanes 4–7: fractions 7–10, respectively; and M, molecular weight marker. (B) A representative gel resulting from an SDS-PAGE analysis of the proteins fractionated by ion exchange column chromatography. The proteins were passed thorough an ion exchange column and eluted with a linear gradient of 150–1,000 mM NaCl. Lane 1: applied sample; lane 2: column flow-through; lanes 3–10: fractions 16–23, respectively; and M, molecular weight marker. Original gel images of S2-A and S2-B are shown in S2-C and S2-D, respectively. (TIFF) [file pone.0204160.s002.tiff]

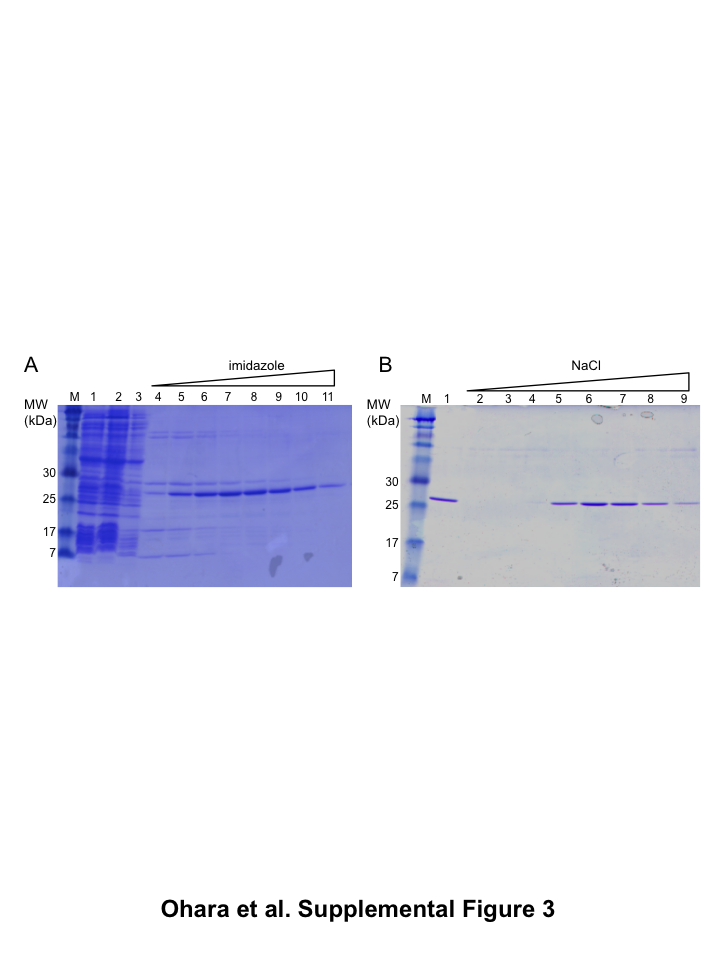

Supplement: S3 Fig — (A) A representative gel resulting from an SDS-PAGE analysis of the proteins fractionated by a HIS-trap column. Recombinant E. coli expressing rFull-MDP1 were lysed by sonication and centrifuged. The supernatant was then loaded onto a His-Trap column in the presence of 10 mM imidazole and eluted by 300 mM imidazole. Lane 1: lysates after disruption of the bacteria; lane 2: applied supernatants of bacterial lysates; lane 3: column flow-through; lanes 4–11: fractions 16–23, respectively; and M, molecular weight marker. (B) A representative gel resulting from an SDS-PAGE analysis of the proteins fractionated by ion exchange column chromatography. The rFull-MDP1 purified by heparin column chromatography was further purified by CM Sepharose column chromatography. The proteins were eluted with a linear gradient of 100–1,000 mM NaCl. Lane 1: applied sample after heparin column purification; lane 2: column flow-through, lanes 3–8: fractions 14–19, respectively; and M, molecular weight marker. Original gel images of S3-A and S3-B are shown in S3-C and S3-D, respectively. (TIFF) [file pone.0204160.s003.tiff]

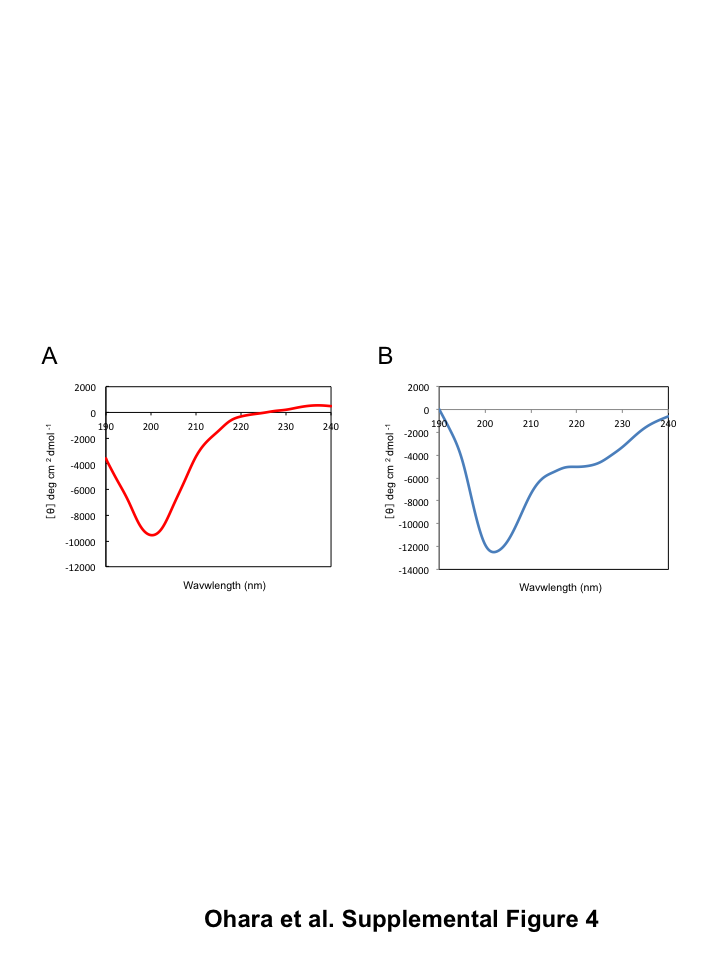

Supplement: S4 Fig — (A) CD spectra of rFull-MDP1 purified through acid extraction. (B) CD spectra of rFull-MDP1 purified by the refined method without acid extraction. Proteins were resolved in phosphate buffer (pH 7.0) containing 150 mM NaCl. (TIFF) [file pone.0204160.s004.tiff]

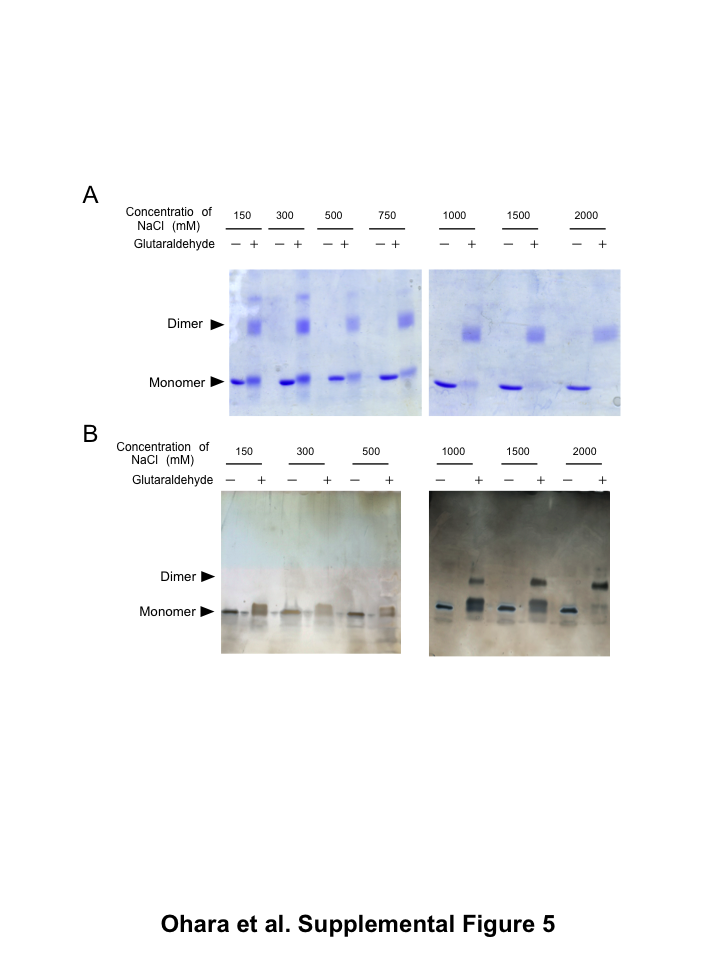

Supplement: S5 Fig — The proteins were cross-linked at various concentrations of NaCl and fractionated with SDS-PAGE. The gels were stained with CBB (A) and silver staining (B). Original gel images of S5-A and S5-B are shown in S5-C and S5-D, respectively. (TIFF) [file pone.0204160.s005.tiff]
